# Do psychological and behavioral factors classified by the West Haven-Yale Multidimensional Pain Inventory (Swedish version) predict the early clinical course of low back pain in patients receiving chiropractic care?

**DOI:** 10.1186/s12891-016-0933-y

**Published:** 2016-02-12

**Authors:** Andreas Eklund, Gunnar Bergström, Lennart Bodin, Iben Axén

**Affiliations:** Institute of Environmental Medicine, Karolinska Institutet, SE-171 77 Stockholm, Sweden; Research Department, Spine Center of Southern Denmark, Hospital Lillebaelt, Institute of Regional Health Research, Østre Hougvej 55, DK-5500 Middelfart, Denmark

**Keywords:** Low back pain, MPI-S, Psychological and behavioral factors, Clinical course, Chiropractic

## Abstract

**Background:**

To investigate if psychological and behavioral factors (as determined by the Swedish version of the West Haven-Yale Multidimensional Pain Inventory, MPI-S) can predict the early clinical course of Low Back Pain (LBP).

**Methods:**

MPI-S data from patients (18–65 years of age) seeking chiropractic care for recurrent and persistent LBP were collected at the 1^st^ visit. A follow-up questionnaire was administered at the 4^th^ visit. The predictive value of the MPI-S subgroups Adaptive Copers (AC), Interpersonally Distressed (ID) and Dysfunctional (DYS) was calculated against the subjective improvement at the 4^th^ visit and clinically relevant difference in pain intensity between the 1^st^ and 4^th^ visit.

**Results:**

Of the 666 subjects who were included at the 1^st^ visit, 329 completed the questionnaire at the 4^th^ visit. A total of 64.7 % (AC), 68.0 % (ID) and 71.3 % (DYS) reported a definite improvement. The chance of “definite improvement”, expressed as relative risk (95 % CI) with the AC group as reference, was 1.05 (.87–1.27) for the ID and 1.10 (.93–1.31) for the DYS groups, respectively. The DYS and ID groups reported higher values in pain intensity both at the 1^st^ and the 4^th^ visit. The proportion of subjects who reported an improvement in pain intensity of 30 % or more (clinically relevant) were 63.5 % AC, 72.0 % ID and 63.2 % DYS. Expressed as relative risk (95 % CI) with the AC group as reference, this corresponded to 1.26 (.91–1.76) for the ID and 1.09 (.78–1.51) for the DYS groups, respectively.

**Conclusions:**

The MPI-S instrument could not predict the early clinical course of recurrent and persistent LBP in this sample of chiropractic patients.

**Trial registration:**

Clinical trials.gov; NCT01539863, February 22, 2012.

**Electronic supplementary material:**

The online version of this article (doi:10.1186/s12891-016-0933-y) contains supplementary material, which is available to authorized users.

## Background

Low back pain (LBP) is a highly prevalent [1, 2] and costly [3, 4] condition considered to cause more disability than any other disorder in the world [5]. The vast majority of people will experience LBP at some point in their life and about two-thirds will experience recurrences [4]. The individual course of LBP may follow a number of different trajectories [6–8] and rather than the common categories of acute, sub-acute or chronic [1] LBP could be described as a long-term recurrent condition. In about 90 % of patients suffering from LBP, no underlying spinal pathology or red flags can be identified, and their LBP is classified as non-specific [4].

Due to the limited knowledge concerning the etiology of LBP, a multitude of treatments has been developed. At best, interventions have shown moderate effects in treatment outcome [9]. One possible reason for not finding highly effective interventions for LBP is the heterogeneity of the condition [4]. A recent literature review suggests that subgrouping patients according to genetic predisposition, psychological and activity related factors holds much promise and may be a suitable way to tailor treatments to yield better treatment outcomes [10], identified in previous research as a priority [11–13].

Psychological [14, 15], behavioral [16] and social factors [17] have been associated with the transition from sub-acute to chronic pain [18–21] and the bio-psycho-social model has become the leading theory in the management of LBP [17, 22–24]. The West Haven-Yale Multidimensional Pain Inventory (MPI) is an instrument that has been widely used to measure and capture the chronic pain experience from the cognitive-behavioral perspective [25]. The MPI instrument has been used to derive clinically meaningful clusters/subgroups [26] named Adaptive Copers (AC), Interpersonally Distressed (ID) and Dysfunctional (DYS) which have been shown to be reliable, valid and sensitive to changes in treatment outcomes [27, 28]. These subgroups have been used to investigate a number of chronic pain conditions including neck pain and LBP [29–31], temporomandibular disorders [32], headaches [33], fibromyalgia [34] and cancer pain [35].

Attempts have been made to define psychological and behavioral factors that predict treatment outcome in chiropractic patients, but the results have been inconclusive [36–40]. In a series of articles titled “The Nordic Subpopulation Program” a number of prognostic factors and their relation to treatment outcome have been investigated as a means of subgrouping patients. One of the most distinctive findings was that outcome at the 4^th^ visit was strongly predictive of the long-term outcome at 3 and 12 months [41]. These results have been replicated in chiropractic LBP populations in Norway [41], Finland [42] and Sweden [43]. Why some patients appear to respond better to treatment is not known, and further investigations are warranted. Psychological and behavioral factors may be what differentiate these patients.

The natural course of LBP has been studied and the results suggest that for a majority of patients a rapid reduction in pain occurs during the first few weeks following an acute episode [44–47]. However many patients do not recover completely and show little further improvement past 3 months [47–49]. The MPI-S subgroups have been shown to have different natural courses of LBP where the ID and DYS subgroups are more likely to have more persistent pain than the AC group [30]. In the chiropractic setting it is possible to predict long-term improvement and non-improvement by the 4^th^ visit [41]. If such a prediction was possible even earlier in the course of treatment (at the first visit) for those with a poor prognosis, extra resources might be allocated or a different approach altogether might be chosen. If the MPI-S subgroups were predictive of short-term (at 4^th^ visit) improvement they might also show similar properties long-term (at 3 and 12 months).

The aim of this study was to investigate if subgroup assignment at the 1^st^ visit using the MPI-S instrument predicts the short-term clinical course among patients with recurrent and persistent LBP receiving chiropractic care.

## Methods

### Study design

In this prospective multicenter outcome study data were collected during the inclusion phase of an ongoing randomized controlled clinical trial (RCT) in a multi-center setting described in detail elsewhere [50].

### Objectives

The objective of the study was to investigate if MPI-S subgroup (AC, ID, and DYS) assignment at the 1^st^ visit among patients with recurrent and persistent LBP receiving chiropractic care could predict the short-term clinical course using the following outcomes:

1. A definite improvement at the 4^th^ visit (primary outcome).
2. A clinically relevant reduction of pain intensity at the 4^th^ visit (secondary outcome).

### Patients and setting

A total of 40 chiropractors were recruited to collect data on consecutive patients seeking treatment for LBP. The clinicians were known to the research team as they had successfully collected data in a previous research project [51] and were accustomed to integrating clinical research into their daily practice. The clinics were located across Sweden. Patients of working age with recurrent (previous episodes the past year) [52, 53] and persistent (more than 30 days of pain the past year) [54] LBP with no indication of serious spinal pathology were recruited. Patients who were pregnant, did not pay for the treatments themselves or had chiropractic treatment less than 3 months ago were excluded. See Table 1 for a summary of the inclusion and exclusion criteria.

**Table 1:** Criteria for eligibility

Inclusion criteria	Low back pain with or without leg pain. 18–65 years of age. Episodes of low back pain previous year. Pain >30 days previous year. Access to mobile phone. Able to send SMS.
Exclusion criteria	Specific spinal or systemic pathology needing treatment elsewhere. Pregnancy. Chiropractic treatment less than 3 months ago. Treatment paid by third-party.

### Data collection

The subjects were screened for eligibility during the 1^st^ visit and a questionnaire with demographic and baseline data (including MPI-S) was administered by the clinician or the receptionist. As the data for this study were collected during the screening phase of an RCT, only some demographic data were collected at this stage (age was collected at the RCT inclusion visit and could therefore only be specified for those subjects who were eligible for the RCT). A follow-up questionnaire was administered (by the same clinician or receptionist) at the 4^th^ visit irrespective of when it occurred. If the patient reported a definite improvement by the 2^nd^ or 3^rd^ visit, the follow-up questionnaire was administered at this visit. Only the patients who reported a definite improvement went on to be included in the RCT. For this study all subjects who responded to the 4^th^ visit questionnaire and had a complete MPI-S dataset were included. A detailed description of the inclusion process of the RCT can be found in a recently published study protocol [50].

### Dependent variables

The primary outcome measure was the self-rated improvement measured at the 4^th^ visit. The patients were asked to rate their perceived improvement on a five-step ordinal scale (*1 = Definitely worse, 2 = Probably worse, 3 = Unchanged, 4 = Probably improved, 5 = Definitely improved)*. Previous research has used the answer as a dichotomous variable defining improvement only if the subject answered “definitely improved” to avoid overestimation of a positive treatment outcome [41–43]. Dichotomizing the variable in this way have been associated with clinically relevant outcomes at 3 and 12 months post treatment and in this study the variable was used in the same way to conform to previous research. As an alternative analysis the primary outcome was also analyzed untransformed with the ordinal scale to capture the variation within the material. The follow-up period was not pre-determined as it was up to the chiropractors to schedule the visits according to their clinical judgment and the patients’ preferences. However, in a previous study, the 4^th^ visit (during a course of chiropractic treatments) occurred within 2 weeks in 42 % of the cases and between 2 and 4 weeks in 29 % of the cases [42]. Therefore it was likely that the follow-up period for the vast majority of the subjects in this study would be within 4 weeks.

As a secondary outcome, the difference in pain intensity between the 1^st^ and 4^th^ visit on the NRS-11 scale was also analyzed. Pain intensity is a commonly reported outcome in LBP research [55] and has been suggested as a standard outcome by the NIH Taskforce [56]. Change scores (difference between baseline and follow-up) of at least two points or 30 % (for chronic pain patients) have been found to be clinically relevant [55, 57]. The data were thus analyzed as a dichotomous variable divided into a minimal improvement of 30 % and less than 30 %. The main reason for choosing change score rather than using an absolute pain score was to allow for comparison with the subjective global improvement measure. Change scores also compensate for the likely differences in baseline values of pain intensity of the subgroups inherent in the clustering procedure. Therefore choosing 30 % change allows for a fair comparison of the clinical course between subgroups. An alternative analysis was performed with pain intensity as a dichotomous variable divided into an improvement of minimum 2 points and less than that.

### Independent variable

Data regarding the subjects’ psychological and behavioral profile were collected during the 1^st^ visit (as part of the screening questionnaire) using the MPI-S instrument. The MPI-S is based on the cognitive-behavioral conceptualization of pain and is made up of 34 items resulting in 8 scales; 5 assessing psychological dimensions (Pain Severity (PS), Interference (I), Life Control (LC), Affective Distress (AD), Support (S)) and 3 assessing behavioral dimensions (Punishing Responses (PR), Solicitous Responses (SR), Distracting Responses (DR)) [25]. From these eight scales three clusters or subgroups are derived, each with a particular set of characteristics [27, 58, 59]. The AC subgroup is characterized by low pain severity, low interference with everyday life due to pain, low life distress, high activity levels and high perception of life control. This subgroup is considered to have the most favorable prognosis and the least long-term sickness absence [30]. The DYS subgroup may be considered as the opposite end of the spectrum with high pain severity, marked interference with everyday life due to pain, high affective distress, low perception of life control and low activity levels. Thus, this is the cluster with the worst prognosis with the most long-term sickness absence [30]. The ID subgroup has been characterized by deviating scores mainly in the behavioral dimensions with low levels of social support, low levels of solicitous and distracting responses from significant others and high scores on punishing responses compared to the DYS and AC patients. These subgroups were used as the predictive variable in the model.

### Data analysis

The subgroups were formed from the eight original scales using centroid vectors from a validated sample [27]. These centroid vectors have been used successfully in a previous publication to characterize 4 different patient populations with LBP [60].

The dichotomized outcome for perceived improvement was analyzed using (robust) modified Poisson regression to determine the chance (expressed as a relative risk) of a definite improvement as well as a clinically relevant reduction of pain intensity at the 4^th^ visit stratified according to the three cluster structure from the initial screening visit.

The patient’s expectation of improvement at the 1^st^ visit was thought to be a possible confounding factor. Expectation was measured during the 1^st^ visit on an 11 point numerical rating scale asking “How great do you think the chance is that your pain will improve considerably?” with answer options ranging from “No chance (0)” to “Great chance (10)”. This single item question was modified from a previously validated question used to measure the expectations of return to work [61].

Outcomes were analyzed without and with “patient’s expectation of improvement” from the 1^st^ visit included in the regression model to control for confounding and interaction.

### Ethics, consent and permissions

The study has been conducted according to good clinical research practice and the guidelines of the Helsinki declaration. All subjects have signed an informed consent form at the 1^st^ visit where they agreed to take part in the trial. The project has been approved by the local ethical research committee at the Karolinska Institutet: 2007/1458-31/4.

## Results

### Subjects

**Fig. 1:**
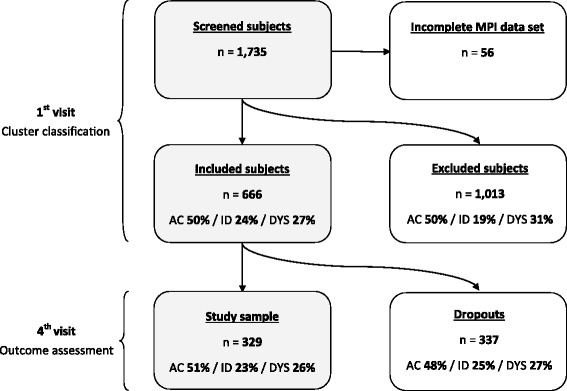
Flow chart. Legend: MPI, The Multi-dimensional Pain Inventory; AC, adaptive coper; ID, interpersonally distressed; DYS, dysfunctional

At the 1^st^ visit a total of 1,735 subjects were screened for eligibility. Fifty six subjects did not complete the MPI-S questionnaire and were excluded. Six-hundred and sixty-six subjects fulfilled the inclusion criteria and were enrolled in the study. At the 4^th^ visit, 329 (45.6 %) subjects completed the follow-up questionnaire (and made up the study sample). Figure 1 describes the inclusion procedure and the distribution of the MPI-S clusters throughout the process.

### Descriptive data

A comparison between the dropouts and the study sample revealed a few differences, however on the whole the groups were similar. Subjects in the study sample were more likely to be female (63.2 % vs 53.4 %) and were more likely to have additional neck and thoracic pain with a previous duration of >30 days in the preceding year (33.7 % vs 24.3 %). Data on age and activity limitation (using the Roland Morris Disability Questionnaire) were collected later in the inclusion process of the RCT, therefore only 156 and 159 subjects had such data. A detailed description and comparison of the study sample and the dropouts can be found in Table 2.

**Table 2:** Baseline data for study sample and dropouts

Variable		Study sample (*n* = 329)	Dropouts (*n* = 337)
Number of subjects, %		49.4	50.6
Age ^A^, mean (n)		44.5 (156) ^A^	- ^A^
Female, % (n)		63.8 (210)	53.4 (180)
Neck and/or thoracic pain ≤ 30 days, % (n)		28.3 (93)	27.3 (92)
Neck and/or thoracic pain >30 days, % (n)		33.7 (111)	24.3 (82)
Thigh, lower leg and low back pain, % (n)		25.2 (83)	23.7 (80)
Living alone, % (n)		0.9 (3)	13.4 (45)
Never visited chiropractor before, % (n)		49.5 (163)	51.3 (173)
Takes medication for their pain, % (n)		21.6 (71)	- ^B^
EQ5D, mean (SD)		.67 (.21)	.66 (.23)
RMDQ ^A^, mean (n; SD)		5.44 (159; 4.22) ^A^	-
Patients perceived health in general, % (n)	Perfect	4.9 (16)	4.1 (13)
	Very good	32.8 (108)	32.5 (103)
	Good	42.2 (139)	41.0 (130)
	Fair	16.1 (53)	18.6 (59)
	Poor	3.6 (12)	3.8 (12)
Patient expectation of improvement 0–10, mean (SD)		8.03 (2.06)	7.56 (2.33)

^A^ Variable was only available for the subjects enrolled into the RCT; ^B^ Data were recorded at the 4^th^ visit; RMDQ, Roland Morris Disability Questionnaire

For the study sample, descriptive data for the individual subgroups (AC, ID and DYS) could, as expected, differentiate the subpopulations. Statistically significant differences could be observed on all variables except; “neck and thoracic pain ≤ 30 days/>30 days”, “previous visit to chiropractor” and “patient’s expected chance of improvement”. The data for the individual clusters are presented in Table 3.

**Table 3:** Baseline data for the study sample (subjects who completed the 4^th^ visit with a complete MPI-S dataset, *n* = 329)

Variable		AC (*n* = 167)	ID (*n* = 75)	DYS (*n* = 87)	p
Number of subjects, %		50.8	22.8	26.4	-
Female, % (n)		58.1 (97)	60.0 (45)	75.9 (66)	.02 ^C^
Age, mean (n)		44.4 (78) ^A^	43.7 (35) ^A^	45.1 (43) ^A^	.87 ^B^
Neck and/or thoracic pain ≤ 30 days, % (n)		29.9 (50)	25.3 (19)	27.6 (24)	.75 ^C^
Neck and/or thoracic pain >30 days, % (n)		30.5 (51)	34.7 (26)	39.1 (34)	.39 ^C^
Thigh, lower leg and low back pain, % (n)		19.2 (32)	28.0 (21)	34.5 (30)	.02 ^C^
Living alone % (n)		.6 (1)	2.7 (2)	.0 (0)	-
Never visited chiropractor before, % (n)		52.7 (88)	45.3 (34)	47.1 (41)	.50 ^C^
Takes medication for their pain, % (n)		12.0 (20)	30.7 (23)	32.2 (28)	<.01 ^C^
EQ5D, mean (SD)		.77 (.11)	.57 (.24)	.57 (.24)	<.01 ^B^
RMDQ ^A^, mean (n; SD)		4.09 (79, 3.77) ^A^	7.00 (35, 3.66) ^A^	6.60 (45, 4.68) ^A^	<.01 ^B^
Patients perceived health in general, % (n)	Perfect	7.8 (13)	1.3 (1)	2.3 (2)	<.01 ^C^
	Very good	47.0 (78)	18.7 (14)	18.4 (16)	
	Good	36.7 (61)	42.7 (32)	52.9 (46)	
	Fair	7.2 (12)	33.3 (25)	18.4 (16)	
	Poor	1.2 (2)	4.0 (3)	8.0 (7)	
Patient expectation of improvement 0–10, mean (SD)		7.97 (2.21)	8.05 (1.96)	8.12 (1.86)	.86 ^B^
MPI-S scales:					
Pain Severity 0–6, mean (SD)		2.74 (.98)	3.87 (.94)	4.28 (0.79)	
Interference 0–6, mean (SD)		2.08 (1.03)	3.61 (1.03)	4.00 (.83)	
Life Control 0–6, mean (SD)		4.13 (.91)	2.70 (.88)	2.86 (.87)	
Affective Distress 0–6, mean (SD)		1.94 (1.08)	3.68 (.90)	3.63 (.93)	
Support 0–6, mean (SD)		3.98 (1.50)	2.95 (1.39)	5.22 (.76)	
Punishing Responses 0–6, mean (SD)		.59 (.76)	2.18 (1.52)	.93 (1.04)	
Solicitous Responses 0–6, mean (SD)		2.43 (1.16)	1.79 (.97)	4.06 (.99)	
Distracting Responses (DR), mean (SD)		2.73 (1.38)	2.11 (1.17)	3.68 (1.09)	

AC, Adaptive Coper; ID, Interpersonally Distressed; DYS, Dysfunctional; ^A^ Variable was only available for the subjects enrolled into the RCT; ^B^ One way Anova for overall difference; ^C^ Chi^2^ test for overall difference

### Primary and secondary outcomes

The proportion of subjects with a definite improvement at the 4^th^ visit was highest in the DYS subgroup whereas the proportion of subjects with a clinically relevant pain reduction was highest in the ID subgroup. When the differences were expressed as relative risks the estimates were small and not statistically significant. The potential confounder, “patient’s expectation of improvement”, did not influence the estimates and was therefore excluded from the final model. The results are presented in Table 4. The alternative analysis of the primary outcome as an ordinal variable using multinomial logistic regression did not change the conclusion and these results were omitted from the report. The results of the alternative analysis of the secondary outcome where pain intensity was computed as a dichotomous variable (where improvement was defined as a reduction of pain intensity by 2 scale steps or more), were similar to the results of the primary analysis and were omitted from the report (all analyzes omitted from the report are available on request from the corresponding author). On average the AC subgroup had significantly lower absolute scores for pain intensity both at the 1^st^ and 4^th^ visit compared to the ID and DYS groups. Absolute values of pain intensity (as cumulative percent) for each subgroup at the 4^th^ visit have been reported in a table as an Additional file 1.

**Table 4:** Self-rated improvement and pain intensity (unadjusted estimates)

Variable	AC (*n* = 167)	ID (*n* = 75)	DYS (*n* = 87)
Self-rated Improvement:			
Definite improvement at 4^th^ visit, % (n; p-value ^C^)	64.7 (108; .56 ^C^)	68.0 (51; .56 ^C^)	71.3 (62; .56 ^C^)
Chance of definite improvement at 4^th^ visit, expressed as relative risk ^A^(95 % CI; p-value)	Ref	1.05^A^ (.87–1.27; .61)	1.10^A^ (.93–1.31; .28)
Pain intensity:			
Pain intensity at 1^st^ visit 0–10, mean (SD; p-value ^B^)	4.47 (1.94; <.01 ^B^)	6.51 (1.97; <.01 ^B^)	6.70 (1.64; <.01 ^B^)
Pain intensity at 4^th^ visit 0–10, mean (SD; p-value ^B^)	2.47 (1.85; <.01 ^B^)	3.57 (1.90; <.01 ^B^)	3.92 (2.14; <.01 ^B^)
Difference in pain intensity between 1^st^ and 4^th^ visit, mean (SD; p-value ^B^)	2.01 (2.30; <.01 ^B^)	2.93 (2.11; <.01 ^B^)	2.78 (2.35; <.01 ^B^)
Reduction of pain intensity of 30 % or more between 1^st^ and 4^th^ visit, dichotomized, % (n; p-value ^C^)	63.5 (106; .39 ^C^)	72.0 (54; .39 ^C^)	63.2 (55; .39 ^C^)
Chance of a reduction of pain intensity of 30 % or more between 1^st^ and 4^th^ visit, dichotomized, expressed as relative risk ^A^ (95 % CI; p-value)	Ref	1.13 ^A^ (.82–1.57; .45)	1.00 ^A^ (0.72–1.38; .98)

AC, Adaptive Coper; ID, Interpersonally Distressed; DYS, Dysfunctional; ^A^ Adjustment for “patient’s expectation of improvement” meant only very small changes in the estimates; ^B^ One way Anova for overall difference between the MPI-S groups; ^C^ Chi^2^ test for overall difference

## Discussion

This is the first study to investigate if psychological/ behavioral profiles classified according to the MPI instrument can predict the short-term clinical course of persistent and recurrent LBP in a chiropractic primary care population. The main strength is the large sample and the use of valid instruments, thus the data are considered robust and reliable.

Our results suggest that the MPI-S subgroups could not predict short-term clinical course at the 4^th^ visit, despite previous research showing MPI-S to have predictive properties concerning long-term sick leave in chronic LBP populations [30]. In the study from Bergström et.al [30] they used absolute scores for sick leave as well as change scores for health related quality of life and found significant differences for the absolute scores but not the change scores. The DYS and ID groups reported significantly higher absolute values in pain intensity both at the 1^st^ and the 4^th^ visit which is in line with the previous study [30]. The DYS and ID groups also reported the largest difference in pain levels between the 1^st^ and 4^th^ visit as well as the largest proportion of subjects with a clinically relevant difference. This indicates that the MPI-S instrument has worked well to classify the subjects into subgroups with significant differences of mean absolute values at the 4^th^ visit; however the change scores of subjective improvement and clinically relevant pain intensity failed to reflect a difference between the subgroups. Although the difference of absolute scores in pain intensity was an important finding the main focus of this article has been to investigate self-reported global improvement, thus change scores were the most appropriate choice of outcome.

The results show that this population is clearly affected by psychological and behavioral distress (MPI-S), however similar to other studies on chiropractic patients the clinical course in this population does not differ significantly between the MPI-S subgroups [36–40]. The study population is a self-selected sample which may have resulted in a selection bias for individuals with a more favorable psychological profile, possibly with a higher degree of self-efficacy. As self-efficacy was not measured this will have to remain a speculation. Another possibility may be that the clinical encounter itself affects psychological factors and although the treatment is mainly physical, it inevitably includes strategies aimed at affecting non-physical factors [40]. Field et.al [40] found that patients’ mean scores of self-efficacy, fear avoidance beliefs and catastrophizing improved within a few days after the initial consultation with a chiropractor. A systematic review found evidence of the long-term effectiveness of SMT for chronic LBP [62] and non-physical effects may very well be important mediators.

The STarT Back screening tool (SBT) is an instrument designed to stratify LBP patients according to modifiable risk factors for poor outcomes [63] and has been shown to improve effect and cost-effectiveness of physiotherapy interventions by using stratified care [64]. A previous study by Field and Newell found that the SBT could not predict the prognosis for LBP patients receiving chiropractic care at 30 and 90 days [36], in line with the results in this study. In Field and Newell’s study one of the dependent variables were pain intensity (NRS-11) and they analyzed both the absolute values and change scores associated with a 2 point reduction, and their results were similar to the results reported here. Similarly Kongsted et al found in a prospective cohort study that the prediction of chiropractic patients’ individual treatment outcome (at 2 weeks, 3 months and 12 months follow-up) was not improved by using the SBT in a stratified treatment approach compared to treatment as usual [65]. Thus, both the MPI-S and SBT seem unable to predict short-term outcome in the chiropractic population. It is important to note that the MPI-S instrument was not designed as a screening tool for fear avoidance, catastrophizing and self-efficacy as the SBT instrument, therefore a direct comparison may be misleading. However it is interesting to see that studies with instruments measuring psychological variables not captured with the MPI-S instrument have arrived at similar conclusions in that they seem to not be associated with treatment outcomes among chiropractic patients. An instrument such as the Örebro Musculoskeletal Pain Screening Questionnaire (ÖMPSQ) that includes a wider range of psycho-social factors may be more sensitive as a prediction tool for LBP in this population [66].

Further, the MPI-S instrument was originally designed to measure the chronic pain experience and is perhaps not suitable for patients experiencing an acute flare-up. Possibly the acute pain disease-pathway affects the subjects differently compared to the chronic pain experience. Previous research has shown that patients who seek chiropractic care in Sweden mostly do so due to an acute episode/exacerbation of LBP (typically with a duration of less than 1 month) [67]; their symptoms are likely to subside given the natural course with a regression towards the mean. Therefore our data may simply illustrate the natural course of the acute flare-up. Nevertheless, research has shown the long-term predictive value of the improvement measure at the 4^th^ visit to have strong correlations with outcomes at 3 and 12 months [41–43].

All subjects in the study sample had recurrent pain and a total of more than 30 days with pain in the previous year. A large proportion (62 %) also had comorbidity with neck and thoracic pain. Therefore chronicity may also have confounded the results as a chronic condition may need more time to respond to an intervention. A long term follow up might reveal different predictive results. Even though the MPI instrument could not predict the short term clinical course, the instrument successfully classified subjects according to psychological and behavioral subgroup profiles. This information may be clinically relevant for other aspects of the patient encounter such as the indication for co-management with other health professionals (e.g. psychologists) for the DYS individuals or involving significant others (spouse, relative or friends) in the treatment plan for the ID individuals. Longitudinal research with a stratified care model in this way could answer these questions.

The study has some limitations. Most important is the possible selection bias from the dropout at the 4^th^ visit (47 % of the study population). Reasons for not completing follow-up may include a fast recovery warranting no further treatment or negative reactions to treatment – both of which may have resulted in discontinuation of the treatment plan. Also the administrative procedures at the clinic may have failed, so that the administration of the 4^th^ visit questionnaire may have been forgotten, contributing to attrition. However, when comparing the descriptive data and the cluster distribution of the dropouts they were similar to the subjects who completed follow-up (results not shown) and the risk of attrition bias is considered to be low. The “perceived chance of improvement” which was slightly higher (and significantly different) in the subjects who completed follow-up may have overestimated the results in a non-differential manner. As in many other outcome studies without a control group, unknown confounders may have biased the results. Therefore randomized controlled longitudinal studies which take into account the long- and short-term effects should be conducted to confirm or reject the results from this study.

As the follow-up data was not recorded at a fixed time point, but during the 4^th^ visit, the data may be confounded by the variability in the time of the follow-up period. As this information was not collected, it is difficult to say to what extent this may have affected the results. However, as previous research have shown the predictive value (regarding long term outcomes at 3 and 12 months [41]) of improvement at the 4^th^ visit (irrespective of when this occurs) it is unlikely that this issue have affected the conclusions to any greater degree.

As some of the descriptive data (age and activity limitation) was recorded at the start of the RCT, they were available only for approximately half of the subjects; which is another limitation of the study.

## Conclusion

The MPI-S instrument could not predict subjective improvement or a clinically relevant reduction of pain intensity at the 4^th^ visit in a population of chiropractic patients with persistent and recurrent LBP.

## Additional file

Additional file 1:
**Cumulative prevalence for each level of pain intensity at the 4** ^**th**^ **visit for each MPI-S subgroup. **(DOCX 14 kb)

## Abbreviations

LBP: Low back pain
NP: Neck pain
MPI-S: Multidimensional pain inventory, Swedish version
MPI: Multidimensional pain inventory
AC: Adaptive copers
ID: Interpersonally distressed
DYS: Dysfunctional
SPSS: Statistical package for the social sciences
ANOVA: Analysis of variance
