# Supplementary material for: Do psychological and behavioral factors classified by the West Haven-Yale Multidimensional Pain Inventory (Swedish version) predict the early clinical course of low back pain in patients receiving chiropractic care?
Source: BMC Musculoskelet Disord. 2016 Feb 12;17:75. doi: 10.1186/s12891-016-0933-y (PMC4751747; doi:10.1186/s12891-016-0933-y)
Supplement: Additional file 1: — Cumulative prevalence for each level of pain intensity at the 4 th visit for each MPI-S subgroup. (DOCX 14 kb) [file 12891_2016_933_MOESM1_ESM.docx]

**Additional file 1:** Cumulative prevalence for each level of pain intensity at the 4^th^ visit for each MPI-S subgroup.

| **Pain intensity (0-10) the past 24 hours (on average)** | **AC (n=167)** | **ID (n=75)** | **DYS (n=87)** | **p** |
| --- | --- | --- | --- | --- |
| 0, % (n) | 13.2 (22) | 4.0 (3) | 3.4 (3) | <.01 **^C^** |
| ≤ 1, % (n) | 32.9 (55) | 16.0 (12) | 10.3 (9) | <.01 **^C^** |
| ≤ 2, % (n) | 55.7 (93) | 26.7 (20) | 29.9 (93) | <.01 **^C^** |
| ≤ 3, % (n) | 77.2 (129) | 50.7 (38) | 47.1 (41) | <.01 **^C^** |
| ≤ 4, % (n) | 88.6 (148) | 70.7 (53) | 63.2 (55) | <.01 **^C^** |
| ≤ 5, % (n) | 92.8 (155) | 85.3 (64) | 79.3 (69) | <.01 **^C^** |
| ≤ 6, % (n) | 158 (94.6) | 93.3 (70) | 80.5 (70) | <.01 **^C^** |
| ≤ 7, % (n) | 98.8 (165) | 97.3 (73) | 95.4 (83) | <.24 **^C^** |
| ≤ 8, % (n) | 99.4 (166) | 98.7 (74) | 98.9 (86) | <.83 **^C^** |
| ≤ 9, % (n) | 100.0 (1) | 100.0 (1) | 100.0 (1) | - |
| ≤ 10, % (n) | 100.0 (0) | 100.0 (0) | 100.0 (0) | - |

**AC**, Adaptive Coper; **ID**, Interpersonally Distressed; **DYS**, Dysfunctional; **MPI-S**, Swedish version of The Multi-dimensional Pain Inventory ; **^C^** Chi^2^ test for overall difference.
